# Development of the Lebanese insomnia scale (LIS-18): a new scale to assess insomnia in adult patients

**DOI:** 10.1186/s12888-019-2406-y

**Published:** 2019-12-27

**Authors:** Souheil Hallit, Hala Sacre, Chadia Haddad, Diana Malaeb, Gloria Al Karaki, Nelly Kheir, Aline Hajj, Rabih Hallit, Pascale Salameh

**Affiliations:** 1 Faculty of Medicine and Medical Sciences, Holy Spirit University of Kaslik (USEK), Jounieh, Lebanon; 2 INSPECT-LB: Institut National de Santé Publique, Epidémiologie Clinique et Toxicologie-Liban, Beirut, Lebanon; 3 Drug Information Center, Order of Pharmacists, Beirut, Lebanon; 4 Research Department, Psychiatric Hospital of the Cross, Jal Eddib, Lebanon; 5 School of Pharmacy, Lebanese International University, Beirut, Lebanon; 6 Life Sciences and Health Department, Universite Paris Est, Paris, France; 7 Faculty of Pedagogy, Holy Family University, 5534 Batroun, Lebanon; 8 Laboratoire de Pharmacologie, Pharmacie clinique et Contrôle de Qualité des médicaments, Pôle Technologie- Santé (PTS), Faculty of Pharmacy, Saint-Joseph University, Beirut, Lebanon; 9 Faculty of Pharmacy, Saint Joseph University, Beirut, Lebanon; 10 Faculty of Medicine, Lebanese University, Beirut, Lebanon; 11 Faculty of Pharmacy, Lebanese University, Beirut, Lebanon

**Keywords:** Insomnia, Scale, Screening, Validation, Lebanon

## Abstract

**Objective:**

To define the development and validation of the Lebanese Insomnia Scale (LIS-18) to be used for the evaluation of insomnia in Lebanese adult patients.

**Methods:**

A first cross-sectional study, conducted between August 2017 and April 2018, enrolled 789 participants (sample 1). A second sample was recruited in May 2018 to confirm the results obtained from the first sample.

**Results:**

Five factors derived from the LIS-18 scale items with an Eigenvalue over 1, explaining a total of 59.64% of the variance (Cronbach’s alpha = 0.821). The first ROC curve, comparing participants with diagnosed insomnia to healthy individuals, showed that the optimal score was seen at a cutoff of 58.00, with a good sensitivity and specificity at this cutoff (93.3 and 88.4%, respectively). A second ROC curve, comparing participants taking drug medication for insomnia vs. those not taking drug, showed that the optimal score was seen at a cutoff of 52.50, with a good sensitivity and specificity at this cutoff (89.5 and 80.0%, respectively). A third ROC curve, comparing participants diagnosed by a physician or taking drug medication for insomnia and healthy control without insomnia drug, showed that the optimal score was seen at 51.50, with good sensitivity and specificity at this cutoff as well (90.0 and 78.10%, respectively). The positive predicted value (PPV) of the LIS-18 score in sample 2 was 93.3%, whereas the negative predicted value (NPV) was 88.4%.

**Conclusion:**

The results demonstrate that the LIS-18 can be used in clinical practice and research to measure insomnia.

## Introduction

Insomnia seems to be one of the most common sleep complaints among people [1]. It affects one’s life negatively especially when it comes to daily functioning, work absenteeism and quality of life [2, 3]. Moreover, it is often correlated to psychiatric disorders, especially depression and anxiety [1, 4]. Its prevalence varies between 6% [5] to 50% [6]. Several factors might be the cause of this wide variation, including but not limited to, the differences in the insomnia definition, the evaluation instruments and geographical settings [7]. Insomnia can be diagnosed via a clinical evaluation [8, 9]. However, health care professionals might be discouraged to conduct such evaluation in their daily practice because of the time restraints, thus, the need for brief and valid self-reporting scales that can facilitate the initial screening [10]. In Lebanon, the prevalence of insomnia reported in recent studies conducted among the general population was 34.5% in one cohort that started in 2014 [11] and 47.1% in a cross-sectional national study that was conducted between 2017 and 2018 [12].

Numerous standardized and validated tools had been established for measuring insomnia intensity, including but not limited to the Athens Insomnia Scale (AIS) [13], the Regensburg Insomnia Scale (RIS) [14], the Insomnia Severity Index (ISI) [15], reflecting the increasing need for assessment instruments in insomnia research. However, each scale screens for one aspect of insomnia, i.e. the RIS to measure the psychological and quantitative aspects of insomnia, the ISI to measure the severity of insomnia symptoms, whereas the AIS includes questions to diagnose insomnia and its next-day consequences. Moreover, the Pittsburgh Sleep Quality Index has 18 questions to be answered by the subject, plus an additional six by a roommate [16], which creates a drawback in the assessment of insomnia in case the person lives alone. Although Arabic versions of some scales were validated in Lebanon [17], none of these instruments was generated in a neighboring country i.e. Middle-East, Arab countries and developing countries and all the cited tools reflect the insomnia perception in the developed countries. In fact, sleeping patterns do vary across cultures despite the levelling tendencies of globalization [18]. Culture is considered as a multifaceted set of behaviors, beliefs, attitudes and practices, which can all influence health and its behaviors [18]. Since sleep is considered a behavior to a certain extent, its pattern is expected molded by culture [18]. Moreover, since these tools cover different aspects of insomnia, we had to use them all to come-up with a final scale covering all of these aspects. The rationale behind the development of a new scale is also based on including the ICD-10 insomnia criteria and giving appropriate importance to subjective sleep difficulty. Subsequently, it was deemed necessary to develop and validate a Lebanese Insomnia Scale (LIS-18) explicitly intended for the Lebanese adult population, taking into account multicultural adaptation procedures and the abovementioned reasons.

## Methods

### Study design and sampling (sample 1)

This cross-sectional study was carried out between April 2017 and August 2018. It enrolled 789 residents of the community randomly selected from Lebanon’s Mohafazat in a proportionate rate. The Mohafazat are divided into Caza (stratum), divided into villages. Two villages per Caza were chosen; households were chosen in each village according to a random sampling technique [19]. All adults living in the household were invited to participate. Those accepting to enroll in the study completed the survey through a face-to-face interview. Excluded were those with self-reported mental illness or dementia, and those who refused to complete the questionnaire. Data collection was performed by study-independent clinical psychologists whose role was to rule out psychiatric problems in each participant. The same methodology was used in previous papers [20–29].

### Minimal sample size calculation

The total number of items taken from different insomnia scales and that was included at the beginning of the analysis, was 30. Thus, a minimal sample size of 30*10 participants (*n* = 300) [30] was needed in order to conduct the validation methods.

### Data collection and measurement

The questionnaire was prepared in Arabic, Lebanon’s native language, and required 15 to 30 min to be completed. It comprised different elements: the first part consisted of sociodemographic features (gender, age, region, marital status, etc.). The socioeconomic level was divided into 3 categories: low, intermediate and high ranging from less than 1000 USD to 2000 USD. The second part included questions hypothesized to be factors associated with insomnia (personal/family history, presence of chronic pain, etc.).

Since cigarette and waterpipe (WP) dependence are known to increase markedly the likelihood of insomnia [31], we decided to use the Fagerstrom scale [32] and the Lebanon Waterpipe Dependence Scale (LWDS-11) [33] to assess dependence to cigarette and waterpipe smoking respectively.

Mental and psychological distress were evaluated using a Lebanese adapted scale the Beirut Distress Scale (BDS-22) [34]. Answers were assessed using a Likert scale from 0 (never) to 3 (very much). The global score was created by adding all the answers for respective items of the score. A score of 25 or more is indicative for high risk of psychological distress [34].

The Hamilton Depression Rating Scale (HDRS), recently validated among the Lebanese population [35], is composed of 21 items; the first 17 items are used to calculate the total score. Answers are based on a Likert scale from 0 (none/absent) to 4 (most severe) spectrum [36].

The 7-item Generalized Anxiety Disorder scale (GAD-7), previously validated in Lebanon [37], allows an adequate assessment for anxiety, it contains 7 questions that ask how often during the last 2 weeks, participants experienced excessive worrying, irritability and difficulty in relaxing [38]. As for the response options, it included “not at all,” “several days,” “more than half the days,” and “nearly every day,” and each one matched respectively 0, 1, 2, and 3.

#### Insomnia scales

When it comes to the diagnosis of insomnia, a systematic search of electronic libraries PubMed, Medline, PsycINFO, Web of Science, and Science Direct was conducted; questions were retrieved from multiple self-reported scales that are used worldwide in order to provide a consistent and trustworthy replication of the person’s quality of sleep. We generated a large pool of items based on interviews conducted with people experiencing insomnia in Lebanon and used their own words to describe their personal experience. We also used five from the most widely used insomnia scales: Athens insomnia scale (AIS), Insomnia severity index (ISI), the Pittsburgh sleep quality index (PSQI), the Regensburg insomnia scale (RIS) and the Medical Outcomes Study Sleep Scale (MOS-SS). It is note noteworthy that the AIS, ISI and RIS scales were validated in Lebanon [39]. The questions were selected after consulting five experts: an Internal Medicine specialist and four community and clinical pharmacists, resulting in a comprehensive questionnaire covering all aspects of insomnia. The role of the experts was to ensure that the chosen questions were reliable, coherent and suitable for the purpose of this study. Questions repeated more than once in different scales or those having similar meaning, were taken only once.

- The AIS is composed of 8 items that self-assesses the intensity of sleep induction, awakenings, sleep duration and quality, etc [40] Scores ≥6 would indicate the presence of insomnia.
- The ISI is composed of 7 items designed to assess the intensity of insomnia and its negative effect on daily work, quality of life and other concerns about sleep disturbances [41].
- The PSQI is a 19-item, self-reporting scale measuring the quality and quantity sleep and their consequences on daily activities [16, 42].
- The RIS includes ten items and is a self-reported tool aiming at measuring the psychological aspect of insomnia [14].
- The MOS-SS is a 12-item, self-reporting instrument for evaluating sleep outcomes [43]. In all scales, higher scores reflect worse sleep problems/sleep quality.

After that, we proceeded with the usual psychometric analyses to retain only a selected number of items best reflecting the construct of interest.

##### Translation process

Translation of all scales questions to Arabic was performed by a certified translator; then a second certified translated the Arabic version back to English. English versions did not differ significantly. A pilot study was run on about 20 subjects -not included in the study- to ensure the understanding of the questions. Few linguistic modifications were done before the launching of the data collection.

##### Clarity of the questionnaire

Flesch reading-ease was calculated to evaluate the ease of reading and clarity of the LIS-18 scale’s questions. The score, obtained directly from the Word document, is based on the average number of syllables per word and the average number of words per sentence. It ranges from 0 to 100, with higher scores indicating an easier document to comprehend [44]. Usually, the average Flesh score obtained for standard writing varies between 60 and 70.

### Sample 2

To validate the results obtained in the first sample, a second cross-sectional study was conducted in May 2018, enrolling participants different from sample 1 (sample 2); this would allow the performance of a confirmatory analysis of the score that was created in sample 1. A face-to-face interview was done with each participant; here, we were able to compare patients with physician diagnosed insomnia versus healthy patients.

### Statistical analysis

Statistical analysis was performed using The Statistical Package for Social Sciences (SPSS) software version 23. The distribution of our sample was normal (verified by the Shapiro Wilk test). The Pearson’s test were used to assess the correlation between two continuous variables. According to Cohen, d = 0.2 would be considered as a small effect size, 0.5 represents a ‘medium’ effect size and 0.8 a ‘large’ effect size [45]. A *p*-value of less than 0.05 was considered significant.

A principal component analysis was initiated to confirm the validity of the construct of the LIS-18 questionnaire in the Lebanese population; we selected to use a promax rotation since the extracted factors were found to be significantly correlated [46]. The Kaiser-Meyer-Olkin (KMO) measurement of sampling adequacy and Bartlett’s sphericity test were appropriate. The factors retained corresponded to Eigenvalues greater than one. In addition, Cronbach’s alpha was used to calculate the reliability of the total score and subscale factors. The intraclass correlation coefficient (ICC) was also used for reliability analysis between insomnia scales. With a Confirmatory Factor Analysis (CFA) done on Sample 2; several goodness-of-fit indicators were reported: the Relative chi square (χ^2^/df), the Root Mean Square Error of Approximation (RMSEA), the Goodness of Fit Index (GFI) and the Adjusted Goodness of Fit Index (AGFI). The value of χ^2^ divided by the degrees of freedom (χ^2^/df) has a low sensitivity to sample size and may be used as an index of goodness of fit (cut-off values:< 2–5). The RMSEA tests the fit of the model to the covariance matrix. As a guideline, values of< 0.05 indicate a close fit and values below 0.11 an acceptable fit. The GFI and AGFI are chi-square-based calculations independent of degrees of freedom. The recommended thresholds for acceptable values are ≥0.90 [47].

## Results

### Study 1

The sociodemographic characteristics of the participants are summarized in Table 1. The mean age of the participants was 37 years (64% females).

**Table 1:** Sociodemographic characteristics of the sample population

		Frequency (%)
Gender	Male	272 (36.0%)
	Female	484 (64.0%)
Living alone	Yes	60 (7.9%)
	No	696 (92.1%)
Education level	Primary	2 (0.3%)
	Complementary	39 (5.2%)
	Secondary	210 (27.8%)
	University	505 (66.8%)
Monthly salary	No income	238 (31.5%)
	Less than 450 USD	75 (9.9%)
	450–1000 USD	237 (31.3%)
	1000–2000 USD	162 (21.4%)
	> 2000 USD	44 (5.8%)
District	Beirut	203 (26.9%)
	Mount Lebanon	460 (60.8%)
	North Lebanon	36 (4.8%)
	South Lebanon	44 (5.8%)
	Bekaa	13 (1.7%)
Marital status	Single	409 (54.1%)
	Married	292 (38.6%)
	Divorced	22 (2.9%)
	Widowed	33 (4.4%)
Smoking	Yes	160 (21.2%)
	No	596 (78.8%)
Waterpipe	Yes	175 (23.1%)
	No	581 (76.9%)
		**Mean ± SD**
Age (in years)		37.00 ± 15.54
Alcohol to sleep		0.12 ± 0.89
Body Mass Index (Kg/m^2^)		25.04 ± 4.34

### Study 2

The mean age of the participants was 33.26 years, 55% of our sample were females and 45.2% were single with low income (42.9%). The majority had a university degree (89.3%) and 59.5% were employee. Fifteen patients (17.9%) were diagnosed by a physician as having insomnia and 22.6% were taking sleep medication. The mean LIS-18 score was 49.95 ± 14.05 with a minimum of 28 and maximum of 81 (median = 46.00) (data not shown). A significantly higher mean LIS score was found in patients with a physician diagnosis of insomnia compared to healthy patients (73.00 vs 44.94; *p* < 0.001).

### Scale’s structure

The questionnaire had a 75.8 readability for Flesch. On all items of the LIS-18 scale, no item has been removed. We run the factor analysis of the LIS-18 scale on the full sample (*n* = 756). Items on the LIS-18 scale converged on a solution of five components with Eigenvalues greater than 1, accounting for a total of 59.64% of the variance (KMO = 0.859, Bartlett’s sphericity test *p* < 0.001). The components generated according to the promax matrix are summarized as follows: Component 1: Sleep thoughts, feelings, physical sensation and behaviors; Component 2: sleep quality and patterns; Component 3: factors related to sleep disturbances; Component 4: daytime sleepiness and impact on daily functioning and Component 5: quantity of sleep. A high Cronbach’s alpha was found for the full scale (0.821), whereas it ranged between 0.694 and 0.794 for the subscales (Table 2).

**Table 2:** Promax rotated matrix of the Lebanese Insomnia Scale items

Factor	Items	Factor 1	Factor 2	Factor 3	Factor 4	Factor 5
I am afraid to go to bed because of my disturbed sleep	RIS 8	.820				
I think a lot about my sleep	RIS 7	.744				
I feel that I have not slept all night	RIS 6	.721				
I wake up from the slightest sound	RIS 5	.657				
Awaken short of breath or with a headache?	MOS-SS 5	.640				
Sleep induction	AIS 2		.846			
Awakenings during the night	AIS 1		.778			
How long did it usually take for you to fall asleep during the past 4 weeks?	MOS-SS 1		.659			
Final awakening earlier than desired	AIS 3		.606			
Total sleep duration	AIS 4		.563			
During the past month, how often have you had trouble sleeping because you feel too hot	PSQI 5 (g)			.794		
During the past month, how often have you hadtrouble sleeping because you feel too cold	PSQI 5 (f)			.772		
During the past month, how often have you had trouble sleeping because you have pain	PSQI 5 (i)			.742		
Take naps (5 min or longer) during the day?	MOS-SS 11				.788	
Snore during your sleep?	MOS-SS 10				.611	
Functioning (physical and mental) during the day	AIS 8				.479	
On the average, how many hours did you sleep each night during the past 4 weeks?	MOS-SS 2					.739
Get enough sleep to feel rested upon waking in the morning?	MOS-SS 4					.698
Cronbach alpha		0.788	0.754	0.718	0.794	0.694
Percentage of variances explained		28.80	10.30	7.72	6.86	5.95

**Component 1:** Sleep thoughts, feelings, physical sensation and behaviors; **Component 2**: sleep quality and patterns; **Component 3:** factors related to sleep disturbances; **Component 4:** daytime sleepiness and impact on daily functioning**; Component:** quantity of sleep

Cronbach alpha for the LIS-18 scale = 0.821

*AIS* Athens Insomnia Scale, *PSQI* Pittsburgh Sleep Quality Index, *RIS* Regensburg Insomnia Scale, *MOS-SS* Medical Outcomes Study Sleep Scale

### Confirmatory analysis

A confirmatory factor analysis was run on sample 2, using the structure obtained in Sample 1. The following results were obtained: the Maximum Likelihood Chi-Square = 418.48 and Degrees of Freedom = 136, which gave an × 2/df = 3.08. For non-centrality fit indices, the Steiger-Lind RMSEA was 0.01 [CI 0.008–0.134]. Moreover, the Joreskog GFI equaled 0.904 and AGFI equaled 0.911.

### Bivariate analysis

The bivariate analysis of the factors associated with the insomnia scales showed that stress, depression and anxiety were positively correlated with insomnia using all the scales, with a medium effect size. The LIS-18 had a better correlation with depression and mental quality of life compared to all three scales (although having a weak effect), whereas it had a better correlation with stress and anxiety than the MOS-SS and the AIS scales but lower than the RIS scale (Table 3).

**Table 3:** Bivariate analysis of factors associated with the different insomnia scales

		LIS-18	RIS	AIS	ISI	MOS-SS
Stress	Correlation coefficient *r*	0.543	0.559	0.500	0.535	−0.450
	***p*-value**	**< 0.001**	**< 0.001**	**< 0.001**	**< 0.001**	**< 0.001**
Depression	Correlation coefficient *r*	0.580	0.560	0.566	0.530	−0.334
	***p*-value**	**< 0.001**	**< 0.001**	**< 0.001**	**< 0.001**	**< 0.001**
Anxiety	Correlation coefficient *r*	0.595	0.621	0.518	0.592	−0.436
	***p*-value**	**< 0.001**	**< 0.001**	**< 0.001**	**< 0.001**	**< 0.001**
Physical quality of life	Correlation coefficient *r*	0.017	0.005	0.017	−0.028	− 0.009
	***p*-value**	0.633	0.887	0.650	0.447	0.808
Mental quality of life	Correlation coefficient *r*	−0.104	−0.085	−0.046	− 0.053	0.150
	***p*-value**	**0.004**	**0.020**	0.213	0.150	**< 0.001**

*AIS* Athens Insomnia Scale, *ISI* Insomnia Severity Index, *PSQI* Pittsburgh Sleep Quality Index, *RIS* Regensburg Insomnia Scale, *MOS-SS* Medical Outcomes Study Sleep Scale

### Intraclass correlation coefficient (ICC) between insomnia scales

A high ICC was found between ISI and RIS (0.875 ranging from 0.856–0.892; *p* < 0.001). Also a high ICC was found between AIS and ISI (0.767 ranging from 0.731–0.798; p < 0.001) (Table 4).

**Table 4:** Intraclass correlation coefficient between insomnia scales

		RIS	AIS	ISI	MOS-SS
RIS	**ICC (CI)**	N/A	0.750 (0.711–0.783)	0.875 (0.856–0.892)	−3.378 (−4.050--2.796)
	***p*-value**		**< 0.001**	**< 0.001**	1.000
AIS	**ICC (CI)**	0.750 (0.711–0.783)	N/A	0.767 (0.731–0.798)	−0.978 (−1.282--0.715)
	***p*-value**	**< 0.001**		**< 0.001**	1.000
ISI	**ICC (CI)**	0.875 (0.856–0.892)	0.767 (0.731–0.798)	N/A	−2.406 (−2.929--1.953)
	***p*-value**	**< 0.001**	**< 0.001**		1.000
MOS-SS	**ICC (CI)**	−3.378 (−4.050- -2.796)	−0.978 (−1.282- -0.715)	−2.406 (− 2.929--1.953)	N/A
	***p*-value**	1.000	1.000	1.000	

*ICC* Intraclass Correlation coefficient, *CI* 95% Confidence interval, *N/A* Not Applicable

*AIS* Athens Insomnia Scale, *ISI* Insomnia Severity Index, *RIS* Regensburg Insomnia Scale, *MOS-SS* Medical Outcomes Study Sleep Scale

### Concurrent validity

The results showed that a higher LIS-18 score was significantly correlated with a higher AIS score (r = 0.678; *p* < 0.001), higher ISI (=0.577; *p* < 0.001), higher PSQI (r = 0.574; *p* < 0.001) and a lower MOSS sleep scale score (r = − 0.671; *p* < 0.001).

### Validity measures

**Fig. 1:**
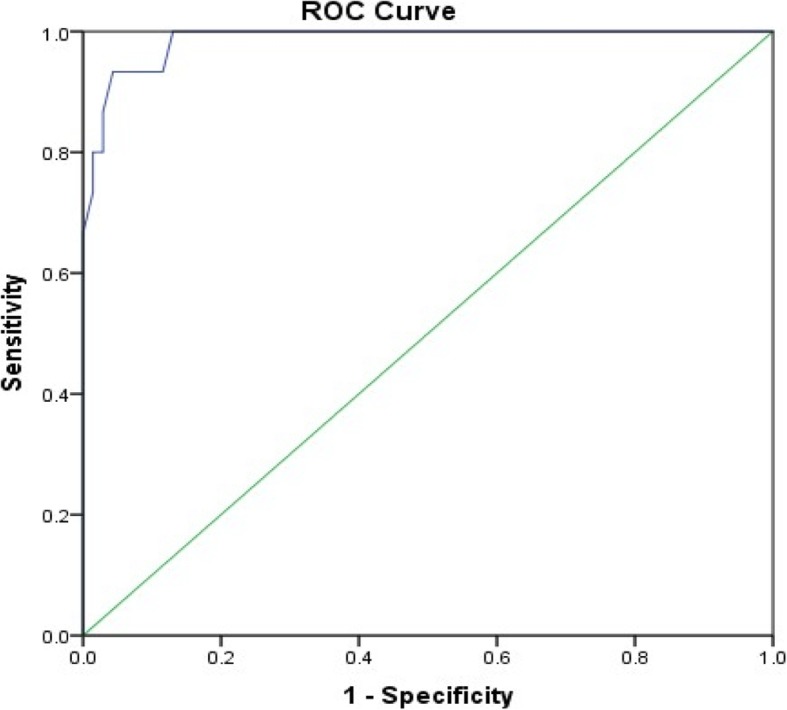
ROC curve of the LIS-18 scale. Patients with physician diagnosis of insomnia vs. healthy control were analyzed. Area under the curve = 0.986 [0.966–1.000] (P < 0.001); at value = 58.00, Se = 93.3% and Sp = 88.4%

**Fig. 2:**
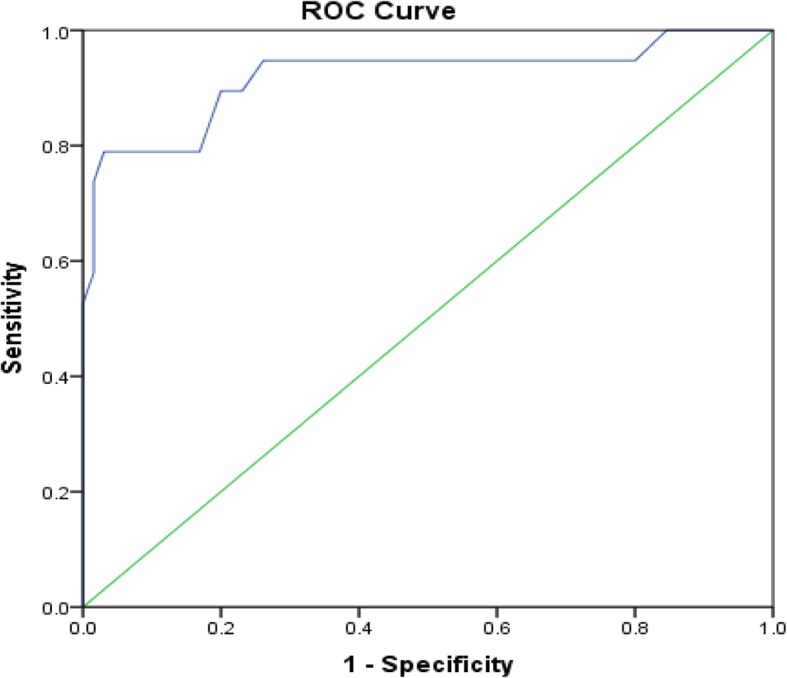
ROC curve of the LIS-18 scale. Patients taking drug medication for insomnia vs. those not taking drug were analyzed. Area under the curve = 0.920 [0.832–1.000] (*P* < 0.001); at value = 52.50, Se = 89.5% and Sp = 80.0%

**Fig. 3:**
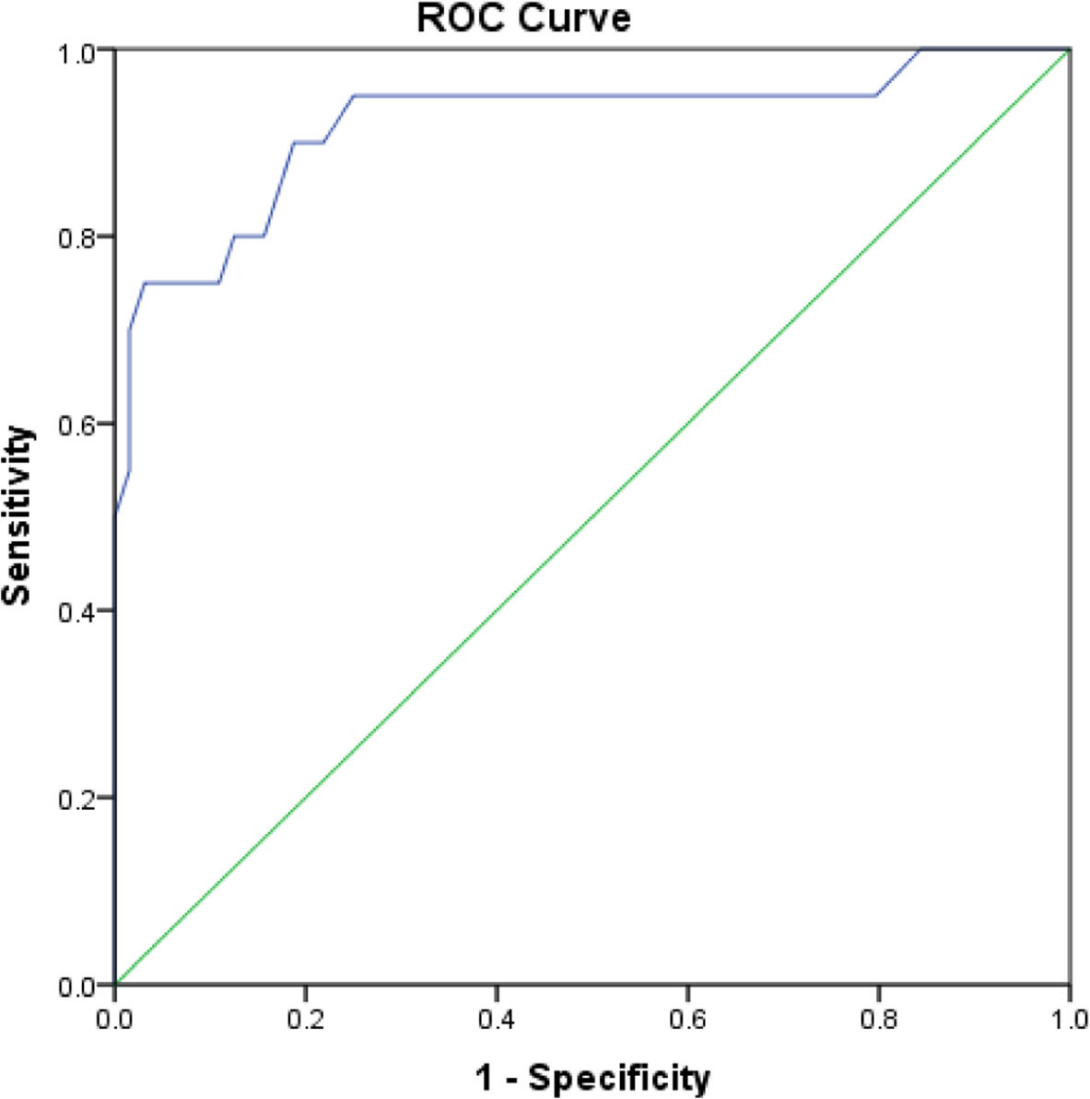
ROC curve of the LIS-18 scale. Patients diagnosed by a physician or taking drug medication for insomnia and healthy control without insomnia drug, were analyzed. Area under the curve = 0.920 [0.836–1.000] (P < 0.001); at value = 51.50, Se = 90.0% and Sp = 78.10%

Three ROC curves of the LIS-18 scale were performed. The first one, comparing participants with diagnosed insomnia to healthy individuals, is shown in Fig. 1. The optimal score that was a cutoff between healthy controls and insomnia patients, was 58.00 (Fig. 1). The sensitivity and specificity were good at this cutoff (93.3 and 88.4%, respectively). The area under the curve was high: 0.986 [0.966–1.000]; *p* < 0.001. The second ROC curve, comparing participants taking sleep medications to those who don’t, were shown in Fig. 2. The optimal score was 52.50 according to the ROC curve analysis (Fig. 2). The sensitivity and specificity were good at this cutoff (89.5 and 80.0%, respectively). The area under the curve was high: 0.920 [0.832–1.000]; *p* < 0.001. The third ROC curve, comparing participants diagnosed by a physician or taking medications for insomnia and healthy control not taking insomnia medications were analyzed (Fig. 3). The optimal score was 51.50 according to the ROC curve analysis (Fig. 3). The sensitivity and specificity were good at this cutoff (90.0 and 78.10%, respectively). The area under the curve was high 0.920 [0.836–1.000].

### The predictive value/face validity

The positive predicted value (PPV) of the LIS-18 score in sample 2 was 93.3%, whereas the negative predicted value (NPV) was 88.4%.

## Discussion

In this study, we were able to create a new scale for insomnia, the Lebanese Insomnia Scale (LIS-18), based on existing scales but adapted to the Lebanese population. Our results suggest that all Arabic versions of the insomnia scales had adequate internal reliability in assessing sleep problems. However, since the ICC of some scales did not correlate with others, this demonstrates the difference between the questions asked in terms of aspects of insomnia that are assessed; it was therefore deemed essential to create a new scale that would regroup all the above insomnia scales’ items and be culturally adapted to the Lebanese population. Thus, the new LIS-18 scale is composed of 18 items, combining all symptoms and aspects of insomnia (physical, psychological, cognitive, emotional and behavioral) with an average completion time of less than ten minutes and an acceptable Flesch reading ease, which makes it an extremely efficient instrument for research and clinical practice.

### Scale development

Scale development is a complex task, based on a clear procedure composed of 3 phases, with a total of 9 steps [48]. The recommended steps for the creation of a new scale according to the international guidelines were followed [48]. In Phase 1, the researcher should state the subject he/she desires to tackle, develops items to be integrated in the questionnaire and determine their validity. The second phase consists of scale construction and includes the pre-testing of questions, the administration of the survey with subsequent item reduction and factor analysis.

In our study, we clearly specified that our aim is to investigate the characteristics of Lebanese people concerning their insomnia symptoms. A “new” instrument was developed since none of the available ones were adapted to the Lebanese population as previously mentioned in the article.

Regarding the factor analysis, results showed that LIS-18 questions were able to tackle all aspects of insomnia (Sleep thoughts, feelings, physical sensation and behaviors; sleep quality and patterns; factors related to sleep disturbances; daytime sleepiness and impact on daily functioning and quantity of sleep). These features make our scale more thorough than the previously validated scales.

The internal consistency of the LIS-18, was found to be very satisfactory (Cronbach’s alpha: 0.82) and comparable to that of the AIS-8 version (α = 0.89) or the AIS-5 version (α = 0.87) [40]. It was higher than that of other instruments used to assess sleep difficulty in clinical settings such as the PSQI [49], the Sleep Problems Scale [50], and the Karolinska Sleep Diary’s sleep quality index component [51].

Moreover, the percentage of subjects correctly identified by the LIS-18 (93.3%) is comparable to the percentage (89%) obtained with the Athens Insomnia Scale [40] and the Pittsburgh Sleep Quality Index [16], showing a good convergent validity. In comparison with clinical diagnosis, the NPV of the LIS-18 was 88.4% (close to 100%); this indicates that virtually the majority of the participants who score less than 62.5 can be reliably considered as not suffering from insomnia. This finding proves the exceptional qualification of the LIS-18 as a screening tool; it allows the identification of persons who do not need further sleep examination in case they obtained a lower score than the mentioned cutoff. On the other hand, the LIS-18 positive predictive value, using the same cutoff for the general population, is fairly decent. Therefore, the new scale can be used in clinical practice to evaluate insomnia-related problems.

### Limitations

The study did not enroll participants suffering from other sleep disorders (i.e. restless legs syndrome, sleep apnea). There might also be an over- or underestimation of the symptoms, which might lead to an information bias. A selection bias is also possible, although we have no reason to believe that it might negatively affect our results. Further studies that overcome these drawbacks and confirm our findings are necessary; in addition, test-retest reliability analysis is also required to consolidate the results.

## Conclusion

In conclusion, the current study results show the new LIS-18 is a valid tool to measure the extent of insomnia in clinical practice. The importance of this scale remains in the fact that it is short, easy to administer and combines all aspects of insomnia. The LIS paralleled positively against other international insomnia scales in terms of consistency, reliability, and validity measures. These assets make it a helpful psychometric tool in sleep research and clinical practice.

## Data Availability

All data generated or analyzed during this study are not publicly available to maintain the privacy of the individuals’ identities. The dataset supporting the conclusions is available upon request to the corresponding author.

## Abbreviations

AIS: Athens insomnia scale
BDS: Beirut Distress Scale
CAS: Central Agency of Statistics
GAD: Generalized Anxiety Disorder
HDRS: Hamilton Depression Rating Scale
ICC: Intraclass correlation coefficient
ICD: International Classification of Diseases
ISI: Insomnia severity index
LIS: Lebanese Insomnia Scale
LWDS-11: Lebanon Waterpipe Dependence Scale
MOS-SS: Medical Outcomes Study Sleep Scale
NPV: negative predictive value
PPV: Positive predictive value
PSQI: Pittsburgh sleep quality index
RIS: Regensburg insomnia scale
